# Problematic Social-Networks-Use in German Children and Adolescents—The Interaction of Need to Belong, Online Self-Regulative Competences, and Age

**DOI:** 10.3390/ijerph17072518

**Published:** 2020-04-07

**Authors:** Sina Ostendorf, Elisa Wegmann, Matthias Brand

**Affiliations:** 1 Department of General Psychology: Cognition and Center for Behavioral Addiction Research (CeBAR), University of Duisburg-Essen, 47057 Duisburg, Germany; sina.ostendorf@uni-due.de (S.O.);; 2 Erwin L. Hahn Institute for Magnetic Resonance Imaging, 45141 Essen, Germany

**Keywords:** social-networks-use disorder, internet addiction, social media use, social networking sites, protective competences, self-regulation, social needs, behavioral addiction

## Abstract

Adolescents nowadays spend much time communicating via social networks. Recent investigations also report a noticeable proportion showing a problematic usage behavior, underlining the importance of better understanding its development and maintenance in young individuals. Theoretical views on Internet-use disorders assume that specific predispositions and needs can contribute to addictive behaviors in interaction with further aspects including Internet-related cognitive biases. This study focuses on vulnerable individuals due to their age and investigates interactions between possible risk (need to belong, NTB) and protective factors (online self-regulative competences, OSRC). Participants (*N* = 466) between 10 and 17 years answered questionnaires assessing social-networks-use disorder symptoms, NTB, and OSRC. Moderated regression analysis revealed significant effects of age, NTB, and OSRC. Three-way interaction was also significant (potentially mainly caused by females), with highest social-networks-use disorder symptoms found for individuals with high NTB and low OSRC, especially when older. With high OSRC, symptoms were significantly lower for both younger and older individuals having high NTB. However, even if NTB was low, older individuals showed high social-networks-use disorder symptoms if their OSRC were low. The results highlight the importance of improving specific competences to prevent problematic usage behaviors, which should be considered in youth-tailored prevention and intervention programs.

## 1. Introduction

Given the easy and almost unrestricted access to social media applications including social networking sites such as Facebook, video/photo sharing platforms such as Instagram or TikTok, and instant messengers such as WhatsApp [1,2], social interactions are largely taking place online. By sharing personally relevant information via text, voicemails, photos, and videos, individuals can communicate with others, express and present themselves, build and maintain relationships, or perceive social support and connectedness [3,4,5,6]. Especially, young people seem to value the possibilities to socially interact via online applications as this can strengthen their sense of belonging [3,7]. Following research on media use of more than 1200 German children and adolescents between 12 and 19 years, 95% stated that they owned a smartphone [8]. In the new reports of EU Kids Online [9], the authors also point out that the majority of young people state that they use the smartphone almost all the time, with adolescents aged between 15 and 16 years spending about twice as much time online as children aged between nine and 11 years. Furthermore, during their leisure time, the usage of the Internet in general, the usage of the smartphone and listening to music were the most important media activities stated by German adolescents in 2018 [8]. Regarding the Internet-usage time, communication accounted for the largest share, followed by entertainment and games, whereby the most frequently used communication applications were WhatsApp, Instagram, and Snapchat [8]. Communicating and staying in contact with others is generally an important motive for using social networks, as highlighted by several authors [10,11,12,13,14,15]. Following Pertegal, Oliva, and Rodríguez-Meirinhos [16], research also identified two important motives for very young individuals: on the one hand obtaining social recognition, and on the other hand belonging to an online community.

Besides several advantages of such applications, research has also critically pointed out that the use of social networks can have undesired and disadvantageous consequences as well. These include, for example, becoming a cyberbullying victim, being threatened by other people, or experiencing problems in everyday life due to an uncontrolled usage [17,18,19]. Based on recent developments in the International Classification of Diseases (11th revision; ICD-11) including the classification of gaming disorder as a disorder due to addictive behaviors, researchers also discuss the problematic use of social networks as potential addictive behavior [20,21]. We therefore use the term social-networks-use disorder (although this term has not been included in classification systems, so far) for the definition of an uncontrolled, problematic use of online-communication applications. This describes the experiences of negative consequences due to, and the diminished control over the use of, social networks, whereby the communicative and social aspect is considered as the key element the users are addicted to rather than a certain device or platform [21,22]. 

With respect to age, being younger was associated with a more problematic social-networks-use [23] and an addictive/problematic smartphone behavior [24,25]. A recent study with a representative sample of German adolescents reported an estimated prevalence of 2.6% for a problematic social-networks-use [26], which is in line with the European-wide prevalence rates ranging from 0% to 2.1% [9]. The results emphasize the relevance of investigating this phenomenon in children and adolescents. They also highlight the importance of investigating potential mechanisms that contribute to the development and maintenance of such behaviors especially in younger ages in order to derive possible implications for prevention measures. Brand and colleagues [27,28] proposed a theoretical model (Interaction of Person-Affect-Cognition-Execution; I-PACE) to specify mechanisms involved in addictive behaviors, such as the problematic use of social networks. The I-PACE model is based on the assumption that specific motives, needs and further personal predispositions including social deficiencies interact with affective and cognitive processes (e.g., Internet-related cognitive biases such as specific expectancies) as well as executive components (e.g., reduced inhibitory control) and experiences of gratification and compensation in the development and maintenance of addictive behaviors. Considering the development of a (potential) social-networks-use disorder, the aforementioned processes may lead individuals to use specific social network services, resulting in experienced gratification and/or compensation (supposedly depending on individual’s addiction stage) and possible reinforcement processes, potentially intensifying a problematic/addictive usage [28]. As one further extension of the development and maintenance of a social-networks-use disorder, Wegmann and Brand [22] specify the addiction process and assume that especially individuals with specific needs (e.g., high need to belong) or social deficits may be vulnerable to developing a problematic use of social networks, since the experience of gratified social needs may reinforce specific expectancies and coping styles, and may lead to the recurring decision to use those applications to strengthen a sense of belonging.

Need to belong in general is considered as a fundamental human motivation [29] and was previously assumed to be a driving motivator for the use of social networks, such as Facebook [5]. The authors propose a dual-factor model of Facebook use including two primary social needs: the need to belong, representing an intrinsic drive to get in touch and connect with others, and the need for self-presentation, corresponding to impression management processes [5]. A study by Beyens, Frison, and Eggermont [30] confirmed the proposed role of need to belong and found that it is associated with an increased Facebook usage. Besides, Martin [31] reported a positive correlation between individual’s need to belong and the frequency of checking social media applications as well as intensity of social-networks-use. Wang and colleagues [32] even found that the need to belong depicts a positive predictor for what the authors called “adolescent smartphone addiction” (note that this term has been criticized recently [33]), and it further moderated the relation between self-esteem and addiction. The results show that the effect of need to belong as a risk factor interacts with further variables, which is consistent with the I-PACE model by Brand and colleagues [28]. Brand and colleagues [28] also highlight that the relevance of predisposing variables, such as usage motives, on the tendency of an additive behavior is related to further affective and cognitive responses such as Internet-related cognitive biases (e.g., specific Internet use expectancies, Internet-related competences). Several studies have already shown that the interaction of those Internet-related cognitive biases and predisposing variables is related to addictive behavior online [2,34]. 

Focusing on the relevance of Internet-related competences, Wegmann, Stodt, and Brand [35], for example, found that self-regulative competences as a subdomain of Internet literacy had a negative effect on the symptom severity of a social-networks-use disorder and, moreover, partially mediated the relationship between psychopathological symptoms and symptom severity. The authors conclude that the ability to regulate one’s own online behavior and to be able to manage time online might be a preventive factor for developing a social-networks-use disorder [35]. This conclusion is consistent with further studies, since Stodt, Wegmann, and Brand [36] also found a preventive effect of self-regulation when considering an unspecific Internet-use disorder. Additionally, Błachnio and Przepiorka [37] concluded that a dysfunction of self-regulation and self-control might be a risk factor for a problematic social-networks-use and van Deursen, Bolle, Hegner, and Kommers [24] found a negative effect of general self-regulation on addictive usage behaviors, but a positive effect of age on self-regulation. This underlines, on the one hand, that self-regulation can be considered as a protective factor preventing a problematic usage, and, on the other hand, implies that especially young individuals seem to have a low level of general self-regulation. Going one step further, online-specific self-regulative competences could play an even more relevant role when investigating problematic social-networks-use in children and adolescents.

**Figure 1:**
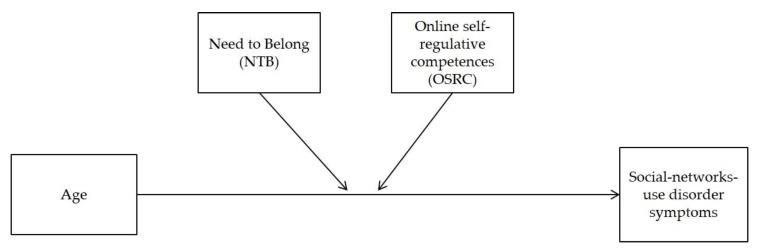
Hypothesized moderated regression model with age, need to belong (NTB), and online self-regulative competences (OSRC) as (moderating) predictors for a social-networks-use disorder.

Based on the aforementioned studies and theoretical assumptions, we argue that there is strong need for investigating not only possible factors in isolation, but especially their interactions in order to better understand the development and maintenance of a problematic social-networks-use. This appears particularly important in order to support prevention and intervention measures especially for young individuals. We hypothesize that the effect of need to belong on symptoms of a social-networks-use disorder is moderated by the ability to self-regulate one’s own online behavior. However, when keeping in mind that the online behavior of children and adolescents is notably changing during childhood and adolescence [9], the interaction effects have to be controlled for a possible age effect. This means that we include age as first predictor to investigate the effect age may have on symptoms of a social-networks-use disorder. We then include need to belong and online self-regulative competences to examine their incremental validity beyond age. Overall, we aim at gaining deeper insights into the protective power of online self-regulative competences by focusing on individuals at risk due to their age and specific needs. Our hypothesized model is depicted in Figure 1. 

## 2. Materials and Methods 

### 2.1. Participants and Recruitment

In total, 466 children and adolescents between 10 and 17 years (*M* = 13.05, *SD* = 1.99) took part in the current study. Of these, 239 were female and 227 were male. The frequency of individual ages was as follows: 10.5% (49 participants) were 10 years old, 18.2% (85) 11 years, 12.2% (57) 12 years, 18.9% (88) 13 years, 13.1% (61) 14 years, 11.4% (53) 15 years, 13.5% (63) 16 years, and 2.2% (10) 17 years. The modal value and the median are both 13 years. Of all participants, 85 stated that they used the Internet actively up to one hour a day, 186 between two and three hours, 105 participants four to five hours, 79 more than five hours a day, and 11 did not report their daily Internet usage time. All participants used at least one social media application including social networking sites, instant messaging services, and photo/video sharing platforms. The most frequently mentioned app was WhatsApp (used by 99.4% of all participants), followed by Instagram (55.4%) and Snapchat (55.2%). Facebook was used by 18.7% of all participants, the Facebook Messenger by 12.9% and Twitter by 15.7%. Besides, 37.1% of the participants mentioned that they used further services, for example Musical.ly (now known as TikTok), YouTube, or Threema. Of all 466 participants, 463 stated to have either their own or a shared smartphone and/or computer. Another three participants reported to not have their own or a shared device but stated that they used at least one social media application. Since the usage of specific applications is the more relevant condition, we decided to include these three participants as well.

The current sample was recruited in 2017 at a local secondary school in a large city in Western Germany within a period of two weeks. Parents and legal guardians gave their informed consent before children and adolescents voluntarily took part. All persons involved (including parents/legal guardians, teaching staff, school administration, and pupils) were informed about the purpose of the study and participants were surveyed in the school premises. The processing of all questionnaires (including those that are not relevant for the current manuscript) took about 40 to 60 min. The study was conducted in accordance with the Declaration of Helsinki and was approved by the local ethics committee of the University of Duisburg-Essen (date: 21st March, 2017).

### 2.2. Instruments

#### 2.2.1. Short Internet Addiction Test Modified for Social-Networks-Use Disorder

In order to measure tendencies of a social-networks-use disorder, a modified version [35] of the short Internet Addiction Test [38] was answered by the participants. This modified version measures subjectively perceived problems in an individual’s everyday life due to the use of online-communication applications, including active (e.g., creating posts/content) and passive use (e.g., browsing/reading content) of social networking sites, (micro-)blogs, and instant messengers. In the course of this study, 4 out of originally 12 items were applied in order to keep the study as short as possible. These four items are considered as key items to represent the scale’s two factors: loss of control/time management and social problems/craving [39,40]. In detail, these were “How often do you find that you spend more time with online-communication applications than you intended?” and “How often do you neglect household chores to spend more time with online-communication applications?” for the subscale loss of control/time management and “How often do you feel preoccupied with online-communication applications when offline, or fantasize about online-communication applications?” and “How often do you choose to spend more time with online-communication applications over going out with others?” for social problems/craving. All items were rated from 1 = “never” to 5 = “very often”. The sum score for the four items ranges from 4 to 20. The overall internal consistency was α = 0.844.

#### 2.2.2. Single-Item Need to Belong Scale

The extent of participants’ need to belong was assessed by applying the Single-Item Need to Belong Scale by Nichols and Webster [41], to which a good reliability and validity is attributed. Here, participants are asked to rate from 1 = “disagree” to 4 = “agree” to what extent the statement “I have a strong need to belong” applies to them. Thus, higher scores indicate a higher need to belong.

#### 2.2.3. Internet Literacy Questionnaire

To measure online self-regulative competences we used the Internet Literacy Questionnaire developed by Stodt and colleagues [42]. The scale assesses four dimensions of individuals’ competences in using the Internet in an adequate way, namely technical expertise, reflection and critical analysis, production and interaction, and self-regulation. The subscale self-regulation consists of five items, which are answered on a 6-point Likert scale ranging from 0 = “strongly disagree” to 5 = “strongly agree”. For the purpose of this manuscript, we only used the subscale self-regulation with an internal consistency of α = 0.752. A mean score was used to test the hypothesis.

### 2.3. Statistical Analyses

We used SPSS 24.0 for Windows [43] for the statistical analyses. To test for bivariate correlations, we calculated Pearson’s correlations, with |r| ≥ 0.10 indicating a small, |r| ≥ 0.30 a medium, and |r| ≥ 0.50 a large effect [44]. To test our hypothesis, we calculated a hierarchical moderated regression analysis with age as first predictor, need to belong in the second step, and online self-regulative competences in the third step, followed by the respective interactions and the three-way interaction in the last step. All independent variables were Fisher’s z-transformed beforehand. Here, standardized beta-coefficients serve as measure of the effect sizes (with similar indicators for a small, medium, and large effect as for correlations). Simple Slopes analyses are calculated for significant three-way interactions.

## 3. Results

### 3.1. Descriptive Values and Multivariate Statistics

Mean values with corresponding standard deviations of all variables are depicted in Table 1. Table 1 also shows bivariate correlations between symptoms of a social-networks-use disorder, age, need to belong, and online self-regulative competences. Social-networks-use disorder symptoms were significantly positively correlated with need to belong and age, and significantly negatively with online self-regulative competences. Need to belong was not significantly correlated with both age and online self-regulative competences, whereas age and online self-regulative competences were significantly negatively correlated. Moreover, age correlated significantly positively with participants’ Internet usage time (*r* = 0.369, *p* < 0.001; *n* = 455 due to missing data) and social-networks-use disorder symptoms also correlated significantly positively with Internet usage time (*r* = 0.465, *p* < 0.001, *n* = 455).

### 3.2. Hypothesis Testing

**Figure 2:**
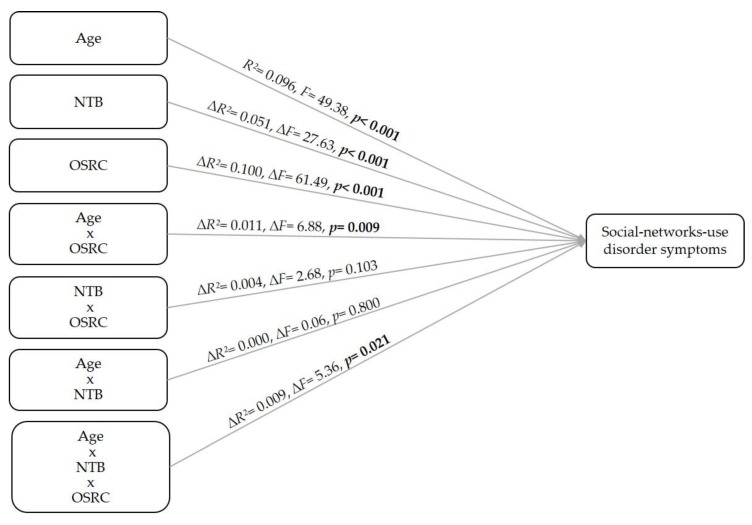
Results of the hierarchical moderated regression analysis with symptoms of a social-networks-use disorder as dependent variable and age, need to belong (NTB), and online self-regulative competences (OSRC) as well as their respective interactions as predictors.

**Figure 3:**
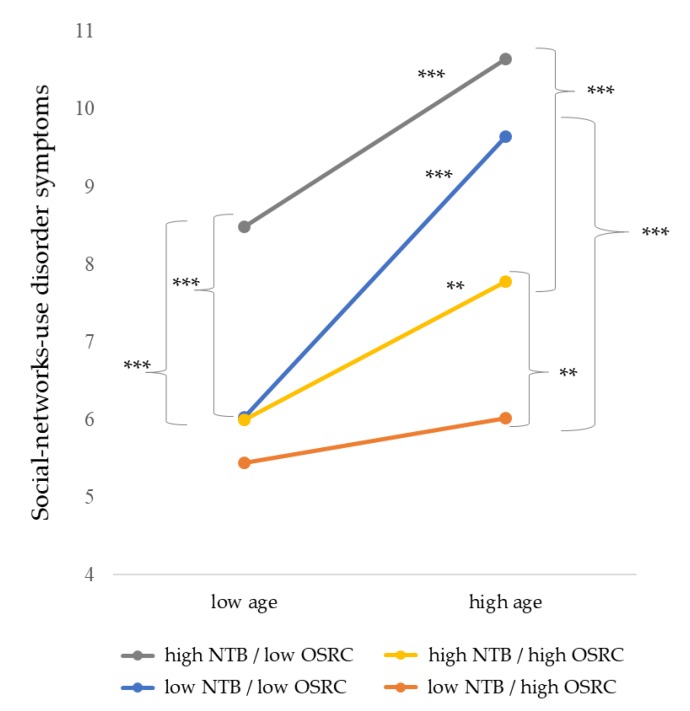
Simple Slopes visualizing the significant three-way interaction between age, need to belong (NTB), and online self-regulative competences (OSRC) in the prediction of social-networks-use disorder symptoms with ** *p* < 0.01, *** *p* < 0.001 and for all significant *t*: 2.65 ≤ *t* ≤ 6.78.

In accordance with our hypothesized model, we calculated a hierarchical moderated regression analysis including age, need to belong (NTB), and online self-regulative competences (OSRC), corresponding interactions and the three-way interaction, in the order as stated. The results revealed significant main effects for age, NTB, and OSRC in the prediction of social-networks-use disorder symptoms (see Figure 2). Furthermore, the interaction effect of age and OSRC was significant (∆*R*^2^ = 0.011, ∆*F* = 6.88, *p* = 0.009), as well as the three-way interaction (∆*R*^2^ = 0.009, ∆*F* = 5.36, *p* = 0.021). The overall model also proved to be significant, explaining 27.1% of the criterion’s variance (*R*^2^ = 0.271, *F* (7, 458) = 24.35, *p* < 0.001) and supporting our assumptions. Investigating the interaction effects in more detail, simple slopes analyses were calculated. The results showed that, especially when older, individuals with high NTB and low OSRC showed the highest tendency towards a social-networks-use disorder (grey line, Figure 3). With higher OSRC, social-network-use disorder tendency was significantly lower in both younger and older individuals with high NTB (yellow line, Figure 3), whereby younger individuals showed even less a social-networks-use disorder tendency than older ones. However, older individuals even had a high social-networks-use disorder tendency when their NTB was low, if they also had low OSRC (blue line, Figure 3). Finally, especially older individuals showed the lowest social-networks-use disorder tendency when they had a low NTB level and also high OSRC. Corresponding statistical values including beta-coefficients are depicted in Table 2. 

### 3.3. Additional Analyses

As an additional analysis, we controlled our results for a potential gender effect. We firstly calculated t-tests for independent samples. We found no significant differences between male and female participants for NTB, OSRC, and age (all *p* ≥ 0.768). For tendencies towards a social-networks-use disorder, we found a significant difference (males: *M* = 6.62, *SD* = 3.14; females: *M* = 8.45, *SD* = 3.68; *t* (458.76) = −5.77, *p* < 0.001, *d* = −0.54). We therefore calculated our hierarchical moderated regression analysis in a second step again separately for male and female participants. For female participants, the effects of age (β = 0.403, *p* < 0.001), NTB (β = 0.214, *p* < 0.001), and OSRC (β = −0.418, *p* < 0.001) on symptoms of a social-networks-use disorder remained significant, as well as the interaction effect of age and OSRC (β = −0.180, *p* < 0.001) and the three-way interaction (β = 0.151, *p* = 0.003). The overall model also proved to be significant and explained 46.0% of the criterion’s variance (*R*^2^ = 0.460, *F*(7, 231) = 28.11, *p* < 0.001). For male participants we also found significant effects of age (β = 0.197, *p* = 0.003), NTB (β = 0.188, *p* = 0.004), and OSRC (β = −0.244, *p* < 0.001), but no significant interaction effect of age and OSRC (β = −0.045, *p* = 0.488) and no significant three-way interaction (β = 0.052, *p* = 0.440). The overall model remained significant, explaining 14.4% of the criterion’s variance (*R*^2^ = 0.144, *F* (7, 219) = 5.25, *p* < 0.001). The subsequently calculated Chow Test comparing both hierarchical moderated regression analyses yielded a significant difference (*F* (8, 450) = 9.37, *p* < 0.001).

## 4. Discussion

In this study, we aimed at contributing to a better understanding of a social-networks-use disorder by considering on the one hand specific needs that can increase the risk of developing a problematic use and on the other hand Internet-related cognitive biases (i.e., self-regulative competences) that may play a protective role. Further, we focused on a specific group of individuals that can be particularly vulnerable to developing a social-networks-use disorder due to their young age (here individuals between 10 and 17 years) and investigated interaction effects between the respective variables. The calculated hierarchical moderated regression analysis revealed that 27.1% of the variance of social-networks-use disorder symptoms were explained by the included variables: age, need to belong, and online self-regulative competences. All variables showed a significant main effect and the three-way interaction was significant as well. Controlling for possible gender effects, the results illustrate that female participants showed higher symptoms of social-networks-use disorder compared to male participants, which is in line with previous studies [45,46]. When calculating the hierarchical moderated regression analysis separately for male and female participants, we found significant main effects of age, need to belong, and online self-regulative competences for both males and females, but the interaction effects only remained significant for females.

The results illustrate that high need to belong may be a vulnerability factor for the development and maintenance of a problematic social-networks-use. The findings support previous reports regarding the important role of need to belong [32] and further strengthen that this fundamental social need is also an important factor for both female and male children and adolescents as potentially contributing to a problematic use of social networks. The findings therefore extend current research, illustrating the relevance of specific needs and motives for using social networks for children and adolescents. It can be assumed that online social networks are perceived as a possible means to feel socially integrated and to gratify individual’s need for belonging [22]. Especially, young individuals may perceive a strong desire to fulfill their need to belong, since they find themselves in an important developmental part of life, in which their respective peer group occupies an important place [30,47,48] and depicts an essential source of experiencing social support [49]. Thus, particularly younger individuals seem to be a vulnerable group that might use social networks in an intense and potentially critical way to gratify their need to be socially connected and accepted. This is in line with the I-PACE model [27,28], assuming that the aim to fulfill personal needs and motives can lead individuals to use specific applications, resulting in gratification and reinforcement processes, which potentially ends up in an uncontrolled or even problematic usage. 

The current results also indicate that high online self-regulative competences are associated with significantly lower symptoms of a problematic use of social-networks in both males and females, underlining the protective power of specific competences and abilities [24,35,36]. These results strengthen previously found empirical results and again broaden current research since the ability to regulate one’s own online behavior appears to be an important competence for very young individuals too. 

When investigating the interaction between both factors (need to belong and online self-regulative competences), we found no significant effect on the symptom severity of a social-networks-use disorder, neither for females, males, nor when taking both together. This result appears unexpected at first sight, but when considering that age had a significantly positive direct effect (for both males and females), and the interaction between online self-regulative competences and age was (except for males) also significant, it becomes apparent that the investigation of interactions between potential risk and protective factors for individuals, and especially for females, in such a young phase of life additionally needs to consider age. As the three-way interaction illustrates, children and adolescents with a high need to belong and low online self-regulative competences had a high problematic usage behavior, especially when older within the age range of 10 to 17 years. Higher online self-regulative competences, in contrast, were related to significantly lower social-networks-use disorder symptoms. Moreover, especially younger adolescents seem to benefit from online self-regulative competences when having a high need to belong level, as they showed even fewer symptoms of a social-networks-use disorder compared to older ones. However, even if the need to belong is low, particularly older individuals seem to need good self-regulative competences in order to reduce the risk of developing social-networks-use disorder symptoms. Since these interaction effects have been observed in the overall sample and in the female sample, but not in the male sample, they may be mainly caused by female participants. However, in accordance with the I-PACE model, interactions between different variables are assumed to contribute to the development and maintenance of problematic behavior generally for both men and women [28]. Thus, theoretically assumed interactions may be valid for both genders, although gender-specific differences can occur. Regarding the for females significant interaction between online self-regulative competences and age, which was not significant for males, one possible explanation could be that female participants reported higher symptoms of problematic behavior compared to male participants and thus the interaction effect may mainly occur along with a certain symptom severity. Moreover, the development of specific competences in such a young phase of life could be assumed to proceed differentially in females and males and thus potential mechanisms may also be different at this age [50]. The development of these competences with regard to age and gender and their relevance as protective mechanisms for a problematic behavior must specifically be addressed in further, preferably longitudinal studies.

Overall, the current results emphasize the importance of improving specific competences, particularly the ability to regulate oneself when using social networks, in order to prevent problematic usage behaviors. They further underline that in a time span of only seven years, young individuals undergo many changes, and that specific needs and also competences can vary in their importance for the development and maintenance of a social-networks-use disorder depending on the respective age. Since we, moreover, found that age and symptoms of a social-networks-use disorder were positively correlated with Internet usage time within our sample, it seems that using the Internet is rapidly increasing in a relatively short age span and is additionally related to the risk of a problematic social-networks-use. This is also in line with the investigation of Smahel and colleagues [9], illustrating that time online is significantly higher in young adults aged between 15 and 16 years, compared to children aged between nine and 11 years. 

Based on the current results and statistics, it appears important to investigate the developmental trends of specific needs as well as competences and strategies especially in youths, in order to better understand respective problematic and uncontrolled behaviors. Self-regulation is likely to be learnable and research has previously pointed out that, when growing older, not only personal interests or goals change, but individuals also seem to be more settled [24,51]. However, loneliness and the need to be socially integrated can also increase over lifetime, probably leading people to use media even more [24,52]. Thus, possible changes and developments over time need to be considered more strongly when investigating the interplay of different factors in the context of problematic online behaviors. In addition, possible differences between male and female children and adolescents should also be kept in mind. Along with changes in people’s communicative behavior, future research also needs to continue addressing the question of when the usage of social networks in order to fulfill personal needs becomes a significant problem. Using specific applications to keep social contact might in general be beneficial, but if individuals, and particularly youths, for example, lose control over their usage and perceive those applications as the only way to feel socially integrated, this might increase negative consequences in their everyday lives. 

To sum up, we strongly recommend that prevention programs should focus specifically on the improvement in self-regulative competences from early on, including children and adolescents, to sensitize for a more functional use of social networks. Individuals should be supported in developing competence to better evaluate an appropriate extent of their social-networks-use, which is meant as an individually suitable amount not causing problems in everyday life. Teaching specific competences in school could help individuals not to satisfy their personal needs exclusively online, but to focus on a more purposeful behavior. Totally restricting youths’ access to the Internet and its various platforms to communicate is certainly not an effective strategy [53], particularly since young individuals use social networks not only to avoid parental control, but also to handle their friendships and to explore new identities in an important stage of life [54,55]. In contrast, finding ways to empower users, especially children and adolescents, seems to be a promising approach, which is also noted by other authors [56]. Besides the communication context, specific competences and needs might also play an important role in other specific Internet-use disorders since there is, at least for German youths, a slow but noticeable increase in using social networks for entertainment and gaming purposes [8]. Thus, the interplay between potential risk and protective factors should also be further investigated in other specific Internet-use disorders.

For the current study, some limitations have to be mentioned. The current sample only includes children and adolescents aged between 10 and 17 years from one school in Germany and is not representative for the whole German population in this age range. The information given is based on self-reports, which did not include questions on participants’ primary time of day to use social networks and other applications. However, this information can be very important for prevention and intervention measures and should be included in future studies [57]. Furthermore, we were not able to assess epidemiological characteristics, such as social class or rural/urban area, but since these aspects can give valuable insights, they should be considered in future studies. Overall, it should be noted that conclusions about possible changes in self-regulation or other specific competences over lifespan from childhood to adolescence are only based on the cross-sectional investigation and should be examined with a longitudinal study. However, the results are a first hint about the relevance of specific needs, specific competences such as online self-regulation, and age on a problematic use of social networks. In addition, younger individuals should also be taken further into account, since certain needs and the use of the smartphone have already been observed in even younger ages. In order to be able to generalize the results, future studies are still needed.

## 5. Conclusions

Childhood and adolescence depict an important phase of life in which communicating and socializing is increasingly realized via social networks. The current study revealed that in particular female children and adolescents aged between 10 and 17 years with high need to belong and low online self-regulative competences showed a high tendency towards a social-networks-use disorder, especially when they were older. High online self-regulative competences were associated with significantly lower symptom severity. Moreover, even if the need to belong was low, older individuals were found to need high online self-regulative competences in order to prevent a problematic usage. Accordingly, prevention and intervention programs should try to improve individuals’ competences, in particular self-regulation, to support individuals in using social networks in a purposeful and appropriate way.

## Figures and Tables

**Table 1:** Mean values, standard deviations, range, and bivariate correlations between the study’s variables.

Variables	*M*	*SD*	Range	1	2	3
1. Social-networks-use disorder symptoms	7.56	3.55	4–20			
2. Age	13.05	1.98	10–17	0.310 ***		
3. NTB	2.58	0.89	1–4	0.202 ***	−0.073	
4. OSRC	3.02	1.20	0–5	−0.374 ***	−0.136 **	−0.077

*Note.* NTB = Need to belong; OSRC = Online self-regulative competences. **p < 0.01; ***p < 0.001.

**Table 2:** Regression coefficients of the hierarchical moderated regression analysis with age, need to belong (NTB), and online self-regulative competences (OSRC) predicting symptoms of a social-networks-use disorder.

Predictors	*B*	*SE (B)*	ß	*t*	*p*
Age	0.51	0.07	0.287	7.00	<0.001
NTB	0.81	0.16	0.203	4.98	<0.001
OSRC	−1.00	0.12	−0.337	−8.11	<0.001
Age × OSRC	−0.18	0.06	−0.115	−2.81	0.005
NTB × OSRC	−0.14	0.13	−0.044	−1.05	0.295
Age × NTB	−0.02	0.09	−0.009	−0.21	0.834
Age × NTB × OSRC	0.17	0.07	0.097	2.32	0.021

*Note*. Significant values depicted in bold.
